# Tissue Engineered Human Skin Equivalents

**DOI:** 10.3390/pharmaceutics4010026

**Published:** 2012-01-06

**Authors:** Zheng Zhang, Bozena B. Michniak-Kohn

**Affiliations:** 1 New Jersey Center for Biomaterials, Rutgers—The State University of New Jersey, Piscataway, NJ 08854, USA; 2 Ernest Mario School of Pharmacy, Department of Pharmaceutics, Rutgers—The State University of New Jersey, Piscataway, NJ 08854, USA

**Keywords:** human skin equivalents, tissue engineering, regenerative medicine, skin grafts, skin models

## Abstract

Human skin not only serves as an important barrier against the penetration of exogenous substances into the body, but also provides a potential avenue for the transport of functional active drugs/reagents/ingredients into the skin (topical delivery) and/or the body (transdermal delivery). In the past three decades, research and development in human skin equivalents have advanced in parallel with those in tissue engineering and regenerative medicine. The human skin equivalents are used commercially as clinical skin substitutes and as models for permeation and toxicity screening. Several academic laboratories have developed their own human skin equivalent models and applied these models for studying skin permeation, corrosivity and irritation, compound toxicity, biochemistry, metabolism and cellular pharmacology. Various aspects of the state of the art of human skin equivalents are reviewed and discussed.

## 1. Human Skin and Skin Barrier

Human skin, as the largest organ in the body, is an anatomical barrier between the internal and external environment; it protects the body from toxic substances, pathogens, and organisms. Human skin is also involved in a number of physiological functions such as fluid homeostasis, thermoregulation, immune surveillance, self-healing, as well as sensory detection [1]. 

Human skin consists of the epidermis and dermis, the epidermis being the outer layer of the skin. The major cells of the epidermis are keratinocytes (90–95%), which proliferate in the *stratum basale* (the deepest layer of epidermis). As the basal keratinocytes proliferate, the “daughter” cells migrate superficially and differentiate, forming the *stratum spinosum* (a layer of spinous cells where keratinization begins), *stratum granulosum* (a granular layer where further differentiated keratinocytes become granular cells that contain keratohyalin granules), *stratum lucidum* (a thin and clear/translucent layer of dead cells; this layer is mostly only seen in palms and soles), and *stratum corneum* (a cornified layer consisting of corneocytes, dead cells lacking nuclei and organelles), respectively. The *stratum corneum* has been proposed to possess a “bricks and mortar” structure, in which the corneocytes represent the “bricks” and the intercellular lamellar lipid bilayers represent the “mortar”; this layer provides the most significant contribution to the permeation barrier properties of human skin [2]. 

Other cells that play a functional role in the epidermis include Langerhans cells (dendritic cells that are present in the *stratum spinosum* and contribute to the immunological responses of the skin), melanocytes (present in the *stratum basale* and produce melanin, which contributes to skin pigmentation and protects the skin from harmful ultraviolet radiation), and Merkel-Ranvier cells (the oval receptor cells present in the *stratum basale* that contribute to sensory reception).

The epidermis layer is bound tightly to the underlying dermis layer via basement membrane at the Dermal-epidermal junction (DEJ). The basement membrane can be divided into *lamina lucida* (the layer closer to the epidermis; this layer is made up of laminin, integrins, entactins, and dystroglycans) and *lamina densa* (a sheet-like structure composed mainly of type IV collagen).

The dermis lies between the epidermis and the subcutaneous tissues, and is composed of the *papillary dermis* and the *reticular dermis*. The *papillary dermis* intertwines with the epidermis, forming rete pegs and rete ridges along the DEJ [3]; as a result, the surface contact area between the epidermis and the dermis increases. The *reticular dermis* is the site of the dense collagen and elastic fiber matrix, and it is the main source of the strength, flexibility and elasticity of the skin. The three major cell types of the dermis are fibroblasts, macrophages, and adipocytes, among those fibroblasts produce collagens, elastic fibers, glycosaminoglycans, and glycoproteins.

Associated with the epidermis and dermis are skin appendages possessing different functions such as sweat glands that regulate body temperature by secreting sweat onto the surface of the skin; sebaceous glands that secrete sebum to moisturize the skin and hair; hair follicles that play an important role in wound healing, as they are a source of keratinocyte proliferation during reepithelialization [4]; arrector pilli muscles that pull hairs straight; and nails that protect distal phalanx and the fingertip. 

Human skin not only serves as an important barrier against the penetration of exogenous substances into the body, but also provides a potential avenue for the transport of functional active drugs/reagents/ingredients into the skin (topical delivery) and/or the body (transdermal delivery). It is proposed that the compounds penetrate the skin using the following pathways [5]:

(a) through *stratum corneum* via **intercellular route** and/or **intracellular route**, and then through the viable epidermis and dermis via partitioning/diffusion;
(b) through the **appendageal pathway**.

Recently, M.S. Roberts and his group proposed a solute structure-skin transport model for aqueous solutions in which permeation rates depend on both partitioning and diffusivity: Partitioning is related to octanol-water partition coefficient, and diffusivity to solute size and hydrogen bonding [6]. 

## 2. Tissue Engineered Human Skin Equivalents

### 2.1. Tissue Engineering and Regenerative Medicine

The most significant and costly problem in healthcare today is the loss or failure of a tissue or organ. In recent years, tissue/organ transplantations of bone, tendon, cornea, heart valve, vein, skin, kidney, heart, lung, liver, pancreas, and intestine, *etc*., have been used clinically to save lives [7]. Tissues and organs that are transplanted within the same person’s body are called autografts; transplants that are performed between two individuals are called allografts. 

In very limited cases, the patients have suitable autografts for transplantation. For allografts, the demand for tissues and organs seriously exceeds the supply, creating a substantial waiting list; moreover, the immune system tends to reject the foreign tissue or organ. Most organ recipients take immunosuppressive drugs for the rest of their lives; further, immunological imbalances caused by transplantation often lead, on the long term, in tumor growth [8]. 

Therefore, there is a significant need for tissue engineering and regenerative medicine approaches aiming at generation of implantable or *in situ* forming tissues and organs. In the tissue engineering approach, cells are seeded in a biodegradable matrix or scaffold. The matrix/scaffold provides adequate three-dimensional structure of the target tissue. While the cells proliferate and differentiate, producing extracellular matrices (ECM), the matrix/scaffold degrades; these processes can eventually result in the formation of functional tissues [8]. Regenerative medicine approaches share the same principles as tissue engineering, but emphasize more the utilization of patients’ only cells e.g., stem cells.

For commercializable clinical applications, reliable cell sourcing and isolation methods play a critical role in providing a sufficient amount of cells for tissue engineering and regenerative medicine approaches. Rheinwald and Green first reported the isolation and serial cultivation of human epidermal keratinocytes from a small biopsy of skin, and the formation of keratinizing colonies from single cells [9]; then, they found that cells growing in the presence of epidermal growth factor (EGF) retained a higher colony forming efficiency [10]. Green *et al.* further reported the method of transferring the intact epithelium sheets using protease Dispase. An estimation of 6000-fold increase in size of total harvest epithelium as compared to that of the initial skin biopsy was made accordingly [11]. Rheinwald and Green’s work provided the method to obtain sufficient keratinocytes for (large-scale) bioengineering of human skin substitutes [12].

For keratinocytes and more importantly for stem cells, the sourced cells need to proliferate and differentiate in appropriate manners; to achieve this result biological growth factors have been utilized. Experiments have shown that platelet-derived growth factors (PDGF) accelerated the normal healing process and up-regulated the genes necessary for wound healing [13]. Another approach to stimulate cell proliferation and differentiation is through *in vitro* cell culture bioreactors that simulate biochemical and mechanical signals and regulate tissue development [14]. Bioreactors have three major components: metabolically active cells that express their differentiated phenotype and produce ECM; polymeric scaffolds providing three dimensional structural supports for cell attachment and tissue growth, being matrices for nutrients/oxygen/waste transport and media for biological and mechanical stimuli; and an environment mimicking *in vivo* conditions where cell-polymer complex can develop into tissues [15]. 

The selection of the scaffolding materials also plays an essential role [16]. Biologically derived, natural macromolecules are exemplified by type I collagen, glycosaminoglycan, and chitosan; the most applied synthetic polymers are aliphatic polyesters based on lactide, glycolide and ε-caprolactone; the concept of creating polymers from naturally occurring metabolites has been intensively explored by Kohn *et al.*, a framework of utilizing tyrosine-derived polymers for tissue engineering applications has been created [17,18]. 

The use of hydrogels as scaffolding materials is another advancement in tissue engineering. Hydrogels have been made using various natural macromolecules including: agarose, alginate, chitosan, collagen, hyaluronan, fibrin, and among others [19]. These hydrogels have high water content and possess structural resemblance to ECM of many tissues. They can be delivered, together with the cells, into the location of tissue defects in a minimally invasive manner [20]; also, nano- and (micro)particles loaded with hydrophilic peptides, proteins, and growth factors can be incorporated into hydrogels, forming novel drug delivery systems [21]. 

In the field of regenerative medicine, Atala *et al.* developed whole organ decellularization technique for the fabrication of bioscaffolds that can be applied for organ bioengineering. Decellularization approach selectively removed only the cellular component of a tissue, while the structure of ECM and vascular network remained [22]. Then, the cultivated cells originated from the patient’s own body were seeded into the bioscaffolds; subsequent culture of the cells-bioscaffolds complexes would result in artificial organs with much lower risk of rejection as compared to a regular organ transplant [23]. 

### 2.2. Need and Criteria for Human Skin Equivalents

Human skin equivalents (HSEs) are bioengineered substitutes composed of primary human skin cells (keratinocytes, fibroblasts and/or stem cells) and components of ECM (mainly collagen). In the last three decades, tremendous efforts have been devoted to the research and development of HSEs, resulting in a number of clinical products and skin models for pharmaceutical and cosmetic companies. In general, HSEs are applied in two major categories: (a) as clinical skin replacements and grafts; and (b) as models for drug permeability tests and toxicity screening. Table 1 summarizes the commercially available HSEs products.

Skin injuries (from burn, accident, acute trauma or chronic wounds, and diseases, *etc*.) can compromise skin barrier and lead to permanent disability or death of the injured person depending on the severity of the wound. Wound dressings that cover the site of the wound allow the reepithelialization, remodeling of granulation tissue, and formation of scar tissue [4]. However, full-thickness skin is not generated in this way. Clinical skin replacements and grafts are in high demand for the treatment of skin injuries: they represent approximately 50% of tissue engineering and regenerative medicine market revenues. In 2009, the potential United States market for tissue-engineered skin replacements and substitutes totaled approximately $18.9 billion, based on a target patient population of approximately 5.0 million. By the year 2019, the total potential target population for the use of tissue-engineered skin replacements and substitutes is expected to increase to 6.4 million, resulting in a potential US market of approximately $24.3 billion [24].

**Table 1:** Summary of commercially available human skin equivalents.

Brand	Company	FDA Approval	Product Description
**1. Clinical skin replacements and grafts**			
Integra^®^ DRT (Dermal Regeneration Template)	Integra Lifesciences	1996	Thin silicone film covering a porous matrix of cow collagen and glycosaminoglycan
Apligraf^®^	Organogenisis	1998	Fibroblasts and collagen combined in dermal matrix onto which keratinocytes are seeded to form an epidermal layer
Epicel^®^	Genzyme	2007	Autologous keratinocytes grown *ex vivo* in the presence of proliferation-arrested mouse fibroblasts
Transcyte^®^/Dermagraft^®^	Advanced Tissue Sciences/Advanced Biohealing	1997/2001	Cryopreserved dermal substitute: human fibroblast seeded onto polymer mesh and cultured *ex vivo*
Orcel^®^	FortiCell Bioscience	2001/2008	Human epidermal keratinocytes and dermal fibroblasts are cultured in separate layers into a Type I bovine collagen sponge
Alloderm^®^/Strattice^®^	LifeCell Co.	None	Acellular cadaver skin matrix
StrataGraft^®^	StrataTech	None	Full thickness skin substitute where a near-diploid human keratinocytes cell line, NIKS, was utilized.
**2. *In Vitro* Permeation and Toxicity Screening Models**			
SkinEthic Rhe (Reconstructed Human Epidermis)	SkinEthic		Human keratinocytes cultured on an inert polycarbonate filter at the air-liquid interface in chemically defined medium
Episkin	SkinEthic		Human keratinocytes cultured on a collagen base which permit terminal differentiation and reconstruction of the epidermis with a functional stratum corneum
Epiderm	MatTek		Neonatal human-derived epidermal keratinocytes (NHEK) cultured to form a multi-layered, highly differentiated model of the human epidermis
EpidermFT	MatTek		Neonatal human-derived dermal fibroblasts (NHFB) and NHEK co-cultured to form a multi-layered, highly differentiated model of the human dermis and epidermis
StrataTest	StrataTech		Full thickness skin model where a near-diploid human keratinocytes cell line, NIKS, was utilized.
Epidermal Skin Test 1000 (EST1000)	CellSystems Biotechnologie GmbH		Reconstructed epidermal model made from primary human keratinocytes; it comprises a fully differentiated epidermis with viable and cornifiedcell layers
Advanced Skin Test 2000 (AST2000)	CellSystems Biotechnologie GmbH		It comprises a dermal equivalent with embedded fibroblasts as a basis and epidermal layer of keratinocytes on top; it is a full thickness model.

The other field of application for HSEs is as models for drug/ingredient permeability testing and toxicity screening. Animal testing of cosmetic ingredients is strictly limited in the European Union; even if no alternative tests are available, the majority of the animal tests are banned [25]. Human cadaver skin and excised animal skin have been traditionally used as topical and transdermal permeation models [26]. Although human cadaver skin replicates *in vivo* permeation performance to some extent, there is a high sample to sample variation. Animal skin, though easily procured, is morphologically different to human skin. Therefore, there is a commercial need for better HSEs that serve as a suitable model for various skin tests. 

For pharmaceutical companies, HSEs can provide specific skin models for diseases such as vitiligo, melanoma, squamous cell carcinoma, psoriasis, and blistering skin disorders as well as models for healthy skin ([27,28,29], for additional information, see Section 2.4 below); this is a unique advantage over the cadaver skin and animal skin specimens.

HSEs can be designed as epidermis only, dermis only, or full thickness (both dermis and epidermis) depending on the application [30]. The ideal HSEs should have differentiated epidermis morphology, appropriate protein expression, similar lipid contents and lipid multi-lamellar structures as those of human skin [31]; the HSEs should be easy to handle and transport, and should be easy to package and ship. When used as a permeation model for drugs/ingredients, the HSEs should produce consistent data that predict the permeation behavior of the tested compounds through human skin. 

### 2.3. Commercially Available Human Skin Equivalents for *in Vivo* Applications: Clinical Skin Replacements and Skin Grafts

Tissue engineered HSEs are the first commercially available and clinically applied organ substitutes [4]. The success was achieved due to major advancements in keratinocyte cell biology, ECM biology, production of collagen scaffolds and polymeric scaffolds, and stem cell biology [32]. 

When skin is wounded, a cascade of biological responses occurs after hemostasis: it begins with immune cells migrating to the site of injury, followed by fibroblasts generating a new tissue matrix; concurrently, reepithelialization and revascularization occur [33]. Without a graft, a full-thickness skin damage of diameter larger than 4 cm is difficult to heal [34]. 

Skin autografts are harvested from uninjured areas and then applied to the excised or debrided areas of the wounded skin of the same individual. Upon application of the skin graft, the capillary network of the wound will merge with the skin graft. However, several problems arise from skin autografts: significant scarring and pigmentation, often the dermis is not replaced, harvesting skin causes a new wound at the donor site, and extensive skin damage cannot be treated using skin autografts [35]. Allogeneic skin grafts harvested from cadavers can also be used; however they face immunogenic rejection and must be replaced [36]. Therefore, bioengineered HSEs could be used to provide a more permanent solution.

Currently, several HSEs are commercially available for clinical applications. Examples include Apligraf^®^, Epicel^®^, Dermagraft^®^, Alloderm^®^, Transcyte^®^, Orcel^®^, Integra^® ^DRT, and Epistem^®^; a few others are currently under clinical trial, one example is StrataGraft^®^ developed by StrataTech Corp. These HSEs can be divided into three major categories: epidermal, dermal, and full-thickness models. 

#### 2.3.1. Epidermal Models

Epidermal skin replacements require a 2–5 cm^2^ skin biopsy from which the epidermis is separated and the keratinocytes are isolated and cultured on top of fibroblasts [36]. Several companies offer epidermal HSEs including Genzyme’s Epicel^®^ (Cambridge, MA, USA). Epicel^®^ is based on the use of a cultured epithelium prepared from autologous epidermal cells on grafts of burn wounds [37]. 

#### 2.3.2. Dermal Models

Dermal skin replacements add greater mechanical stability and prevent the wound from contracting. 

Transcyte^®^, a product made by Advanced Tissue Sciences, Inc. (La Jolla, CA, USA), utilizes seeded neonatal human dermal fibroblasts in a polymeric scaffold that is then cryopreserved, making it a non-living wound covering. Transcyte^®^ has been successfully used as a temporary wound covering after the burn wound has been excised [38]. A derivative of this product, Dermagraft^®^ by Advanced Biohealing, utilizes a biodegradable polygalactin mesh and has shown limited success in diabetic foot ulcer treatment [39].

LifeCell (Branchburg, NJ, USA) developed Alloderm^®^ and Strattice^®^, intact acellular matrices produced from cadaver skin by removing epidermis and the antigenic cellular elements in the dermis. Often, autologous keratinocytes were seeded and cultured on Alloderm^®^ to form epithelium, and the epithelium-Alloderm^®^ structure can be applied for wound and burn closure [40].

A composite skin graft composed of an outer layer of thin silicone film and an inner layer constructed of a complex matrix of crosslinked fibers is marketed under the product name Integra^®^ Dermal Regeneration Template (Intergra^®^ DRT, Integra Life Sciences Corp.; Plainsboro, NJ, USA). Once dermal layer is regenerated, the silicone film on the dermal layer can be removed and replaced with an epidermal autograft. Integra^®^ DRT has been successfully shown to treat burns [41].

#### 2.3.3. Full-Thickness Models

Full-thickness models of HSEs are composed of both epidermal and dermal layers; keratinocytes and fibroblasts, either autologous or allogeneic, are utilized to prepare the bilayer structures [42]. Histological data from Maruguchi *et al*.’s study indicated that keratinocytes cultured for one week on fibroblasts that had been seeded and cultured in a sponge resulted in optimum proliferation and differentiation of keratinocytes and most closely resembled the histology of the epidermis *in vivo.* In addition, the fibroblasts in the dermal layer provided an ample support matrix for the keratinocytes [43]. 

An early example of full-thickness HSEs was bioengineered from a fibroblasts laced collagen lattice covered with epidermal cells [12]. Based on this pioneer work, Organogenesis (Canton, MA, USA) became one of the first tissue-engineering companies and developed the bilayered skin model Apligraf^®^. Apligraf^®^ is made of living allogeneic human skin fibroblasts that are obtained from human foreskins and soluble type I bovine collagen in the form of a gel seeded with keratinocytes. Apligraf^®^ has been used in surgical wound healing [44] and venous ulcers [45], but not with major burns [46].

PermaDerm^®^ (Regenicin, Inc) is a promising new product that can act as a permanent cover of large burns and injuries. It uses keratinocytes and fibroblasts seeded into a collagen sponge [47].

### 2.4. Commercially Available Human Skin Equivalents for *in Vitro* Applications: Models for Drug Permeability Tests and Toxicity Screening

HSEs are used as models for *in vitro* testing [48], and can demonstrate fundamental biological processes of the skin such as the examination of different stimuli that lead to the formation of the epidermis [49], stem cell maintenance [50], wound healing processes [51], the effect of corrosiveness of various chemicals on the skin [52], phototoxicity of substances [53], and toxicity of various chemicals without the need for animal testing. 

HSEs can be utilized to test the permeability of skin to various topically applied cosmetics/personal care agents and drugs. It is important for cosmetic and pharmaceutical companies to have a reliable *in vitro* screening system to test the amount of drugs/active ingredients that permeate into the epidermis [54], dermis, and across the membrane [55]. In recent years, companies such as L’Oreal and SkinEthic have invested heavily in the development of skin models for pharmaceutical, cosmetic and chemical compound testing [27].

The skin permeability barrier that resides in the *stratum corneum*, is composed of a combination of skin lipids, including: ceramides, cholesterol, cholesterol esters and free fatty acids, which are packed into the intercorneocyte space to form multi-lamellar sheets [56]. The presence of the intercorneocyte space has been confirmed through experimentation to be the main route of permeation for most compounds [6,57]. Therefore, in order to create a viable permeation barrier the epidermal differentiation process and the subsequent lipid accumulation and organization need be comparable to that of human skin [28]. 

HSEs for *in vitro* permeation and toxicity applications can be divided into two major categories: namely epidermis-only models and full thickness models. In US, MatTek Corp. developed a serial of HSE models e.g., Epiderm^®^ (human keratinocytes-derived multi-layered model of human epidermis), EpidermFT (human keratinocytes and dermal fibroblasts derived multi-layered model of human epidermis and dermis), MelanoDerm^TM^ skin model (based on co-culture of human keratinocytes and melanocytes), and Melanoma skin model (melanoma cells combined with EpidermFT). Also, StrataTech Corp. (Madison, WI, US) developed a full thickness StrataTest^®^ skin model where a near-diploid human keratinocytes cell line, NIKS, was utilized. 

In Europe, SkinEthic/L’Oreal (France) developed epidermis models SkinEthic^®^ Rhe (Reconstructed human epidermis) and Episkin^®^, and full thickness model RealSkin^®^. Epidermal-skin-test 1000 (EST1000, an epidermis model) and Advanced-skin-test 2000 (AST2000, a full thickness model) were developed by CellSystems Biotechnologie GmbH (Germany). 

**Figure 1:**
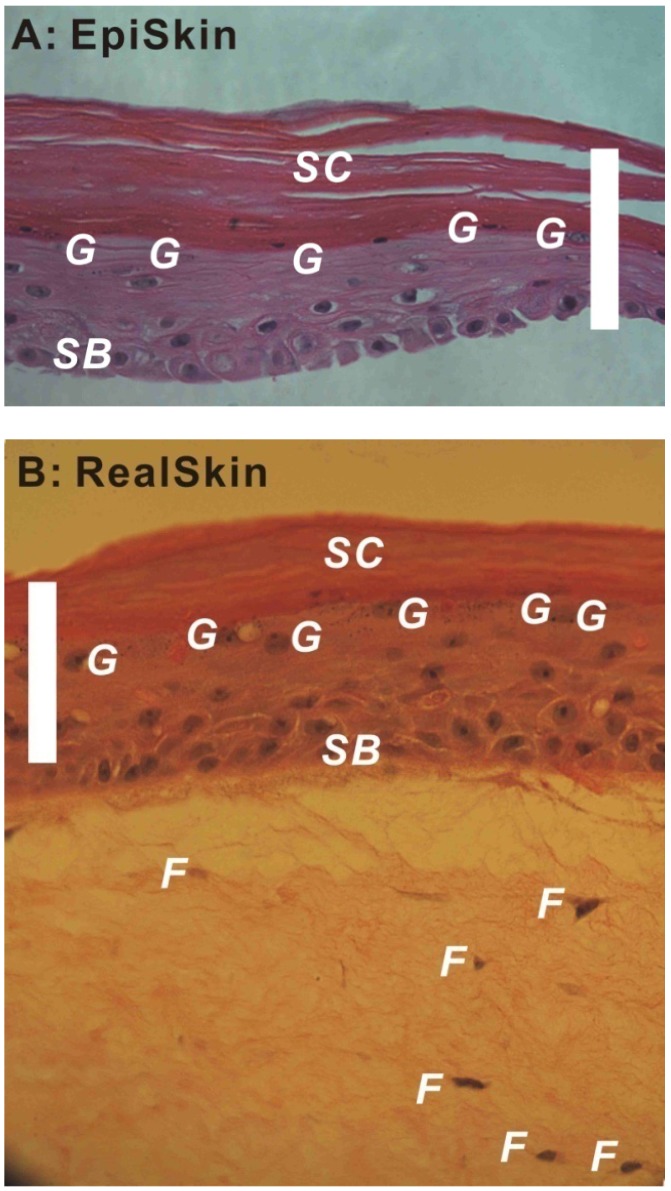
Hematoxylin and Eosin (H&E) stained EpiSkin^®^ (**A**, 40×) and RealSkin^®^ (**B**, 40×) specimens. The HSE samples were received from SkinEthic/L’Oreal (France) and fixed with 4% paraformaldehyde; after dehydration, the specimens were embedded in paraffin, and sectioned into 8 μm thickness. SB: *stratum basale*; G: granular cells; SC: *stratum corneum;* F: fibroblasts (bar = 100 μm).

For these HSEs, the differentiation of epidermis is a key factor determining their barrier properties. Figure 1A,B show the H&E stained images of an EpiSkin^®^ and a RealSkin^®^ specimen, respectively. In both HSE models, the *stratum basale* layer (SB), granular cells (g), and *stratum corneum* (SC) are evident. For RealSkin^®^ specimen, a few fibroblasts (f) are shown, though the typical rete pegs and rete ridges structures along the dermal-epidermal junction in human skin are absent. These images are representative for HSEs of epidermis model and full thickness model, respectively.

The usage of HSEs as alternatives for animal testing must be validated: the batch-to-batch reproducibility of compound permeability must be observed over a long period of time, and multi-laboratory studies need be carried out independently. A number of alternative safety testing methods, where HSEs can be applied as models in the skin corrosivity test and skin irritation test, have been validated by US and international regulatory authorities. These validated alternative methods are listed in Table 2.

**Table 2:** Validated alternative methods where human skin equivalents (HSEs) can be applied as models in the skin corrosivity test and skin irritation test.

Brand of HSE Models	Company	US Regulatory Acceptance/Endorsement by NICEATM-ICCVAM [58]	EU Regulatory Acceptance/Endorsement by ECVAM [59]
**Skin Corrosivity Test**			
EpiSkin^TM^	SkinEthic	OECD Test Guideline 431 accepted in 2004	Commission Regulation (EC) No 440/2008; OECD Test Guideline 431 (April 1998)
Epiderm^TM^	MatTek	OECD Test Guideline 431 accepted in 2004	Commission Regulation (EC) No 440/2008; OECD Test Guideline 431 (March 2000)
SkinEthic^TM^ Rhe	SkinEthic	OECD Test Guideline 431 (meets performance standards 2006)	Commission Regulation (EC) No 440/2008; OECD Test Guideline 431 (November 2006)
EST1000	CellSystems Biotechnologie GmbH	OECD Test Guideline 431 (meets performance standards 2009)	Commission Regulation (EC) No 440/2008; OECD Test Guideline 431 (June 2009)
**Skin Irritation Test**			
EpiSkin^TM^	SkinEthic	OECD Test Guideline 439 accepted in 2010	Commission Regulation (EC) Nr 761/2009; OECD Test Guideline 439 (April 2007)
Epiderm^TM^	MatTek	OECD Test Guideline 439 accepted in 2010	Commission Regulation (EC) Nr 761/2009; OECD Test Guideline 439 (April 2007; modified Skin Irritation Test Method validated in November 2008)
SkinEthic^TM^ Rhe	SkinEthic	OECD Test Guideline 439 accepted in 2010	Commission Regulation (EC) Nr 761/2009; OECD Test Guideline 439 (November 2008)

NICEATM-ICCVAM: The National Toxicology Program Interagency Center for the Evaluation of Alternative Toxicological Methods (NICEATM) and the Interagency Coordinating Committee on the Validation of Alternative Methods (ICCVAM); ECVAM: European Center for the Validation of Alternative Methods; OECD: The Organisation for Economic Co-operation and Development.

### 2.5. Development of Non-Commercial Human Skin Equivalents

Several academic laboratories have developed their own HSEs models. The Stark Group from the German Cancer Research Center (Heidelberg, Germany) has developed a model for *in vivo* study of long-term skin reconstruction and epidermal function. The effect of fibroblasts and microenvironment on epidermal regeneration and tissue function was investigated [50]. The results indicated that: (a) the presence of fibroblasts, and the keratinocyte-fibroblast interactions play a critical role in epidermal tissue regeneration; (b) maintaining a correct microenvironment for epidermal tissue function is important. The HSEs model was also used to study the epidermal homeostasis and to provide experimental conditions for establishing a stem cell niche *in vitro* [60]. 

The Ponec, Bouwstra, and EI-Ghalbzouri groups in The Netherlands have developed the Leiden Human Epidermal (LHE) model, and have utilized it for the evaluation of skin corrosion of chemical compounds in accordance to European Center for the Validation of Alternative Methods (ECVAM) guidelines for testing the corrosive characteristics of chemical compounds [61]. They have developed a method to study HSEs under submerged aqueous conditions to mimic an *in uteru* environment [62]; and they also found that vitamin C has an essential role in the formation of *stratum corneum* barrier lipids [63]. In a recent study performed by EI-Ghalbzouri and Bouwstra groups, the barrier properties of two novel HSEs, the fibroblast-derived matrix model (FDM) and the Leiden epidermal model (LEM), were compared with the full-thickness collagen model (FTM) and human skin [64]. The results demonstrated that the barrier function of the FDM and LEM improved compared with that of the FTM, but all HSEs were more permeable than human skin.

The Morgan group (Boston, Massachusetts) has analyzed the effect of growth factors on cell proliferation for tissue engineering applications [65]. Their findings have indicated that keratinocyte growth factors (KGF) delayed differentiation and induced hyperproliferation. The KGF led to hyperthickening, crowding, and elongation of basal cells without disrupting the barrier function of the epidermis. 

Recently, the Michniak group (the authors’ own group) developed a full-thickness HSE model to serve as a permeation model for topical and transdermal formulations. The dermis of the model consists of human fibroblasts and bovine collagen; on top, keratinocytes are seeded and cultured at air-liquid interface, and differentiate into a highly differentiated epidermal layer. Michniak *et al.* reported that by adding clofibrate, ascorbic acid, and fatty acids into the growth media, lipid composition was improved with values obtained closer to that of human skin. The model overestimated (as all the other HSEs at present do [64,66,67]) the permeation of a number of compounds including caffeine, hydrocortisone, ketoprofen, DEET, malathion, and paraoxon by 2–7 fold when compared to human skin and matched the permeation profiles (*p* < 0.05) of EpidermFT^®^ [68].

## 4. Perspectives

In summary, the skin as an organ has been reproduced using tissue engineering techniques; however, its barrier properties are still low resulting in an overestimation of compound permeability. Most models still lack appendages and a blood supply although melanocytes have been successfully added. The regeneration of hair follicles and sebaceous glands will be a challenge. There is evidence that introducing sebaceous glands into HSEs is a possibility [69]. Another interesting aspect is the regeneration of blood vessels and nerves. The use of stem cells for epidermal differentiation has been explored, but it is far from commercialization and clinical applications*.* Many more studies have to be performed to optimize the current skin models for both clinical use as well as compound permeability testing.
